# Training it Forward: The Role of Embedded Research Fellows in the Network of Scholars Program in Nova Scotia: Comment on "Early Career Outcomes of Embedded Research Fellows: An Analysis of the Health System Impact Fellowship Program"

**DOI:** 10.34172/ijhpm.8653

**Published:** 2024-11-12

**Authors:** Gail Tomblin Murphy, Tara Sampalli, Mark Embrett, Logan Lawrence, Meaghan Sim, Julia Guk, Kaylee Murphy-Boyle, Marta MacInnis

**Affiliations:** Nova Scotia Health Authority, Halifax, NS, Canada.

**Keywords:** Health Services Research, Health Policy Research, Mentorship, Embedded Researchers, Professional Development, Learning Health Systems

## Abstract

Kasaai et al describe the career trajectories of embedded scientists trained through the Health System Impact Fellowship (HSIF), showing that 37% of 2017–2019 HSIF alumni continue as embedded researchers in health systems. These findings suggest that the HSIF program effectively supports career readiness in health services and policy research (HSPR). Similarly, the Network of Scholars (NoS) program, launched post-pandemic in Nova Scotia, mirrors these results, with alumni continuing in embedded roles and mentoring a new cohort of learners from undergraduate to postgraduate levels. NoS has incorporated competencies in quality, project management, and innovation to strengthen training for embedded scientists, aligning with the mandate of the Institute of Health Services Policy and Research. Since 2021, NoS has supported over 100 learners, contributing to over 300 rapid reviews and 100 rapid evaluations addressing top health system priorities while enhancing learner competencies and advancing Nova Scotia’s Learning Health System (LHS) vision.

## Background

 Sustaining learning health systems (LHSs) requires continued investment in health system leaders who are trained to support the culture of using evidence in decision-making, actively promote academic-health system partnerships to generate evidence and facilitate the translation of evidence to policy and practice.^1^ The descriptive analyses by Kasaai et al have contributed to our understanding and considerations of opportunities for careers within health services and policy research (HSPR) by conducting a review of the career trajectories of embedded trainees within the Health System Impact Fellowship (HSIF) – a program designed for launching career readiness in HSPR and fostering LHSs.

 The findings of their study show that all HSIF alumni from cohorts 2017-2019 are currently employed, with 37% working in academia and 46% working in a public or healthcare delivery research role. Of those working in public or healthcare delivery research roles, 22% continue working for the same health service organization (HSO) as their fellowship. The authors further report that 32% of health system impact fellows initially desired to be an embedded scientist at the beginning of their fellowship and currently 37% of alumni hold an embedded scientist position. Although no causal links can be made, the authors’ observations suggest that the HSIF adequately supports HSPR-related career readiness for fellows. As Kasaai et al surmise, the positive trend in HSI fellows working as embedded scholars within HSOs is promising for LHSs in Canada.

 The authors state that the HSI Fellowship program “reinforces the need for PhD and postdoctoral training and curricula to evolve and modernize to prepare trainees for diverse career pathways, bolster preparedness and skills to make an impact in those careers, and to prepare them for leadership roles in learning health systems.”^2^ More recent explorations for essential competencies for this program include the integration of quality, project management, innovation, and data management as part of their learning. This is line with the transformations in health systems nationally and globally where innovation and quality, data and artificial intelligence are playing a central role in the enhancement of access and care.^2-4^

 This commentary describes how an embedded scientists program within the provincial health system in Nova Scotia, namely, the Network of Scholars (NoS)^2,3^ has modernized and expanded their HSPR training environment through the strategy referenced by Kasaai and colleagues.^2^ NoS program implementation was informed by leading models such as HSIF and uniquely designed to accelerate the value of embedded science within the health system and to enhance HSPR career readiness among HSIF (post-doctoral and doctoral) and undergraduate trainees. In this article we first describe the training environment of Nova Scotia Health (NSH), then how it has been applied to the NoS, as well as the successes, challenges, and opportunities that a NoS presents to an LHS. In this response, we demonstrate how the extended training opportunities including a curriculum of innovation and quality offered through the NoS serves to further enhance health system learners’ capabilities as they progress in their education and careers, developing the human capital necessary to support a strong LHS in the provincial health system and a modernized Canadian HSPR training environment first envisioned a decade ago and prescribed by Kasaai and colleagues in their recent article.^2,4^

## Nova Scotia’s Embedded Scientists Training Environment and its Expansion in Training NoS Learners

 Seven HSIF alums (including three authors) are positioned in embedded roles within the provincial health system, of which four had also completed their fellowship training at NSH. These fellows achieved their fellowship goals^5^ of occupying embedded roles within NSH’s Implementation Science Team, while maintaining collaboration with other fellows^6^ and contributing to a LHS using skills enhanced through the HSIF program.^7^ By being embedded within the organization, fellows have the chance to work closely with key stakeholders, policymakers, and practitioners, fostering collaborations that can lead to sustainable changes in healthcare delivery and policy. NSH’s training environment aligns with the goals of the HSIF and provides a unique opportunity for early career researchers to gain hands-on experience within NSH, supporting “real-world impact” that is central to embedded research programs as described by Kasaai et al. This immersive experience helps provide an understanding of the intricacies of the health system, identify areas for improvement, and support the implementation of evidence-based solutions. One of the key aspects of the training environment is mentorship and professional development, as fellows receive guidance and support from experienced mentors who help them navigate the complexities of working within a health system organization.^8^ This mentorship is essential for the fellows to develop their skills, build networks, and enhance their understanding of how research can be effectively translated into practice within the context of a health system.

 With key inputs from embedded HSIF alumni (including ME and MS), and launched by senior leadership (GTM and TS), the NoS was modelled after the HSIF program. It offers structured learning opportunities that provide undergraduate and master’s-level students and graduates with experiential learning within the health system, aimed at developing HSPR-related skills. Thus, the NoS has broadened the embedded training opportunity to encompass all graduate levels of trainees and enhanced the role of mentorship, provided by both embedded scientists and health system leaders, in building a strong LHS. The NoS is a multidisciplinary network of more than 100 learners, fellows, trainees, health system partners, clinician scientists, academic researchers, and patient and community partners with diverse academic backgrounds and skillsets, including nursing, allied health professions, engineering, medicine, computer science, community health, health administration, and epidemiology. These scholars are embedded within the health system to support rapid evidence syntheses and the rapid evaluation of priority implementations in Nova Scotia, directly aligning with the needs of communities and the priorities of the health system. The program provides a flexible onboarding process, enabling participants to engage in experiential learning within the health system, as described elsewhere in this article.

 A foundational element of the NoS program is the Health System Mentorship and Competency Program, where learners gain opportunities to explore and develop their competencies in alignment with their professional goals and health system needs. This mentorship program, which launched in the summer of 2022, matches learners with embedded scientists who act as mentors and share similar interests based on an intake survey. The NoS competency framework was designed based on a review of multiple HSPR-related competency frameworks, including the HSIF,^9^ to provide learners with a well-rounded experience of the health system. Learners work with their mentors to identify one or two key HSPR-related competencies that align with their aspirations, and use these as a guide to align with experiential opportunities, to inform conversations with mentors, and identify other individuals within the NoS that can help support the learner’s development. This approach is not meant to be restrictive—learners generally work on opportunities linked to several HSPR competencies—but helps provide structure and rationale as learners support diverse health system priorities. Self-reflection on development in these competency areas is facilitated through a personalized learning plan and log, helping learners identify changes that may be otherwise overlooked in day-to-day activities and challenges. The Mentorship and Competency Program has shown positive initial outcomes: 100% of learners enrolled in the program during the summer of 2023 reported that they would recommend the program to other learners. Additionally, 100% also reported being satisfied or very satisfied with their mentor and their mentorship experience.

## The Role of NoS in Sustaining Learning Health System Capacity

 While the NoS has only been operating since 2021, we have observed a cycle that has allowed for some degree of sustainability. Students join the team for summer contracts before returning to their studies, recent graduates are given temporary opportunities as research associates to build experience, and NSH continues to host HSI fellows. The NoS prioritizes the personal and career advancement of its participants, and as such, turnover can be seen as a key indicator of the program’s success. The fact that participants move on to other roles or pursue further education reflects the program’s design to foster growth and development, equipping them with the skills needed to advance in their careers. Many learners who participate in the NoS Mentorship and Competency Program move on to advance their studies or careers in other areas of the health system. Since 2022, 45% of these learners who have completed the program (N = 27) have been accepted into health professional training courses, 7% have pursued graduate-level education in HSPR-related fields, and 19% have found leadership or coordination roles within the NSH system. In this way, the NoS supports HSPR-related development for individuals at an earlier stage of their career and training, enhancing the capacity for HSPR in future generations of health system researchers, leaders, and providers, while also supporting a sustainable embedded research program. The NoS’s flexible and continuous intake process has enabled the program to expand as the value of its work becomes increasingly recognized by health system leaders, while program alumni naturally advance in their careers. Much like HSIF, the program serves as a steppingstone for learners seeking health system experience and employment. The NoS presents a valuable training opportunity for learners curious about the intersection between the health system and research but who are too early in their career to avail themselves of opportunities like the HSIF, which is aimed at the doctoral, post-doctoral, or early career research level. The exposure to supporting health system priorities and decision-making gives learners a glimpse into the complexity of the health system and offers a way to review and advance career trajectories while building valuable, transferable skills. While some learners have stayed within the NoS and advanced their roles, others have used their experience to move into other professional programs or other organizations within (and beyond) the Nova Scotian health system (See Figure).

**Figure:**
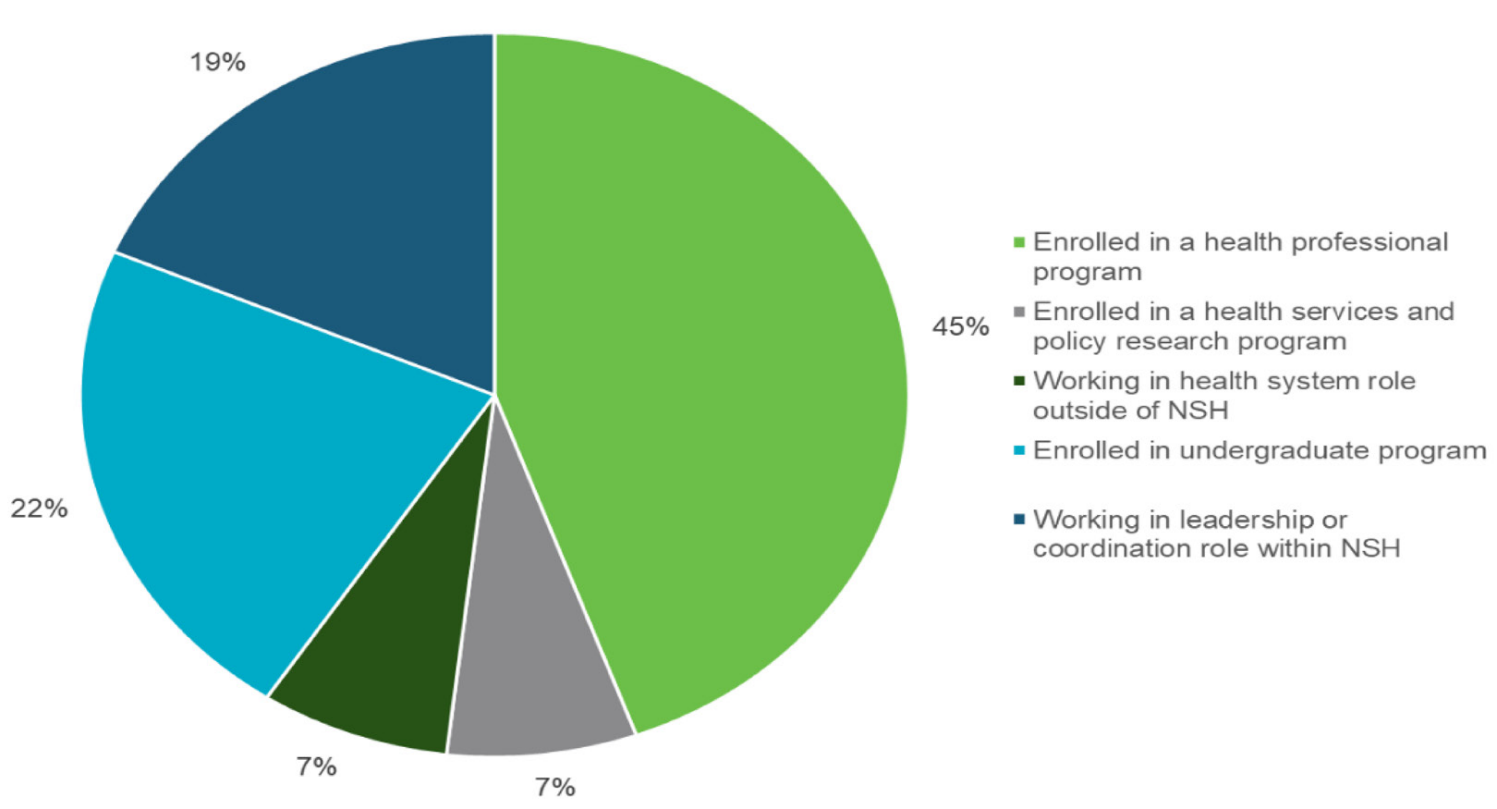
Career and Educational Paths of Network of Scholars Mentorship and Competency Program Alumni (N = 27). Abbreviation: NSH, Nova Scotia Health.

 Similar to the bidirectional benefits of fellows and HSOs participating in the HSIF observed by Kasaai et al,^2^ the NoS supports both the advancement of its members and NSH as an organization. The NoS has been crucial in establishing an integrated innovation and research strategy and LHS within the provincial system, with its members supporting the front-facing priorities of the health system and enhancing internal capacity within NSH to effectively integrate research into practice. Since 2021, the NoS has supported over 300 rapid reviews and over 100 rapid evaluations addressing top health system priorities and challenges to enhance care access and quality for Nova Scotians, from COVID-19 policies and procedures to the integration of novel health professional roles in new settings. The NoS has played a pivotal role in mobilizing evidence to inform implementation, and evaluating key health system initiatives across the province, including mobile primary care units, VirtualCareNS, the YourHealthNS digital navigation service, Virtual Urgent Care NS, and others.^10^ Many of these rapid post-pandemic implementations were made possible by the support of embedded research fellows, who not only conducted rapid evaluations and facilitated learning during the implementation process but also contributed to post-implementation knowledge translation activities. These efforts have helped scale initiatives across the province and disseminate insights more broadly through conference presentations and publications, many of which are currently under review and expected to be published in rapid succession.

 Based on the costs associated with supporting the NoS and the cost savings from rapid reviews and evaluations completed over the past 12 months, the estimated return on investment is approximately $3.7 million. The savings are linked to cost savings linked to the launch and scaling of impactful initiatives within the health system with better access and outcomes for Nova Scotians, and the avoidance of consultant fees for tasks managed by NoS members. NoS members operate within a cost-neutral strategy, where program leaders collaborate with NS Health leaders to secure provincial, federal, and research funding. This funding compensates NoS members and supports the long-term sustainability of the program. By fostering collaboration between NoS members, HSI fellows, and alumni, program leads and NSH leaders, the program strengthens the integration of health systems research and evidence into practice, informing implementation of key innovations to enhance the performance of Nova Scotia’s healthcare system. This collective effort ensures that evidence-informed insights are translated into actionable solutions, ultimately advancing the province’s health system and contributing to better health outcomes for its communities.

## Conclusion

 As demonstrated in the review by Kasaai et al, the HSIF program has played a pivotal role in shaping the career paths of health services and policy researchers, with many fellows continuing to impact the health system in various capacities. Nova Scotia’s provincial health system has built on this success by establishing the NoS, an initiative that extends training to diverse learners. The NoS has become a critical component of NSH’s LHS, offering a dynamic training environment that supports the development of early-career learners through mentorship, leadership training, and practical exposure to health system challenges. Through curated mentorship from senior scientists and by supporting health system initiatives, participants develop their skills that prepare them for future careers in the health system. As we enter a new era, the NoS may have to align itself with fresh opportunities and revise its activities and curricula of learning to best support health system decisions and activities while continuing to provide vital health system exposure for learners, new professionals, and established researchers alike.

## Acknowledgements

 The authors would like to acknowledge Sophia Salmaniw and Janet Rigby of Nova Scotia Health for their contributions to preparation of this paper for publication.

## Ethical issues

 Not applicable.

## Conflicts of interest

 Authors declare that they have no conflicts of interest.
